# Cutaneous Vasculitis and Central Nervous System Infarctions due to Varicella Zoster Virus Vasculopathy in an Immunocompromised Patient

**DOI:** 10.1155/2020/5049627

**Published:** 2020-04-06

**Authors:** Karen Flores Rosario, Katherine C. Michelis, Carol Bjorkman, Faris G. Araj

**Affiliations:** ^1^Department of Internal Medicine, University of Texas Southwestern Medical Center, Dallas, TX, USA; ^2^Division of Cardiology, Department of Internal Medicine, University of Texas Southwestern Medical Center, Dallas, TX, USA

## Abstract

Varicella zoster virus (VZV) infection commonly presents as varicella during childhood, and zoster, later in life. Here, we present a rare and interesting case of VZV infection that manifested with both cerebral and spinal infarctions and cutaneous vasculitis in the absence of a classic vesicular rash in an immunocompromised patient.

## 1. Introduction

Varicella zoster virus (VZV) is a human neurotropic virus that causes chickenpox (varicella) in childhood and herpes zoster (shingles) when reactivation occurs after a period of latency later in life or during decreased immunity [1]. Vasculopathy related to VZV reactivation is due to transaxonal migration of the virus from trigeminal and autonomic ganglia of the head and neck to the cerebral arteries [1]. Complications of VZV vasculopathy include stroke, dissection, aneurysm, and hemorrhage. Interestingly, VZV vasculopathy develops in the absence of rash in 37% of cases [2], and therefore, a high degree of clinical suspicion is needed to make this diagnosis. We report a case of VZV vasculopathy in a heart transplant recipient that highlights how the disease process can involve both the small and large arteries and can present even in the absence of the classic vesicular rash. To our knowledge, this is the first reported case of simultaneous cutaneous, cerebral, and spinal vasculitis secondary to VZV vasculopathy.

## 2. Case Presentation

**Figure 1:**
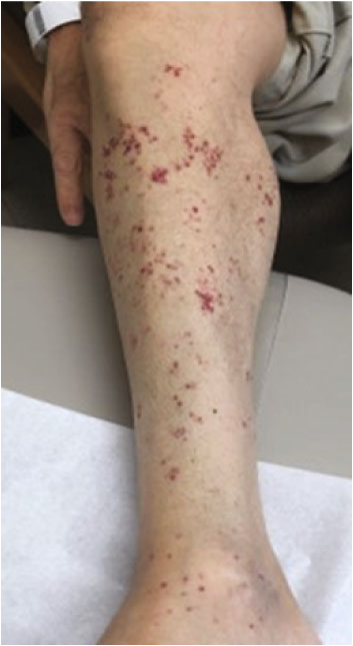
Nonvesicular, purpuric macular rash distributed over the patient's anterior and posterior regions of his right buttock (not shown) and right lower extremity. The rash was not confined to a dermatomal distribution. Pathology of the rash was consistent with leukocytoclastic vasculitis.

A 65-year-old man presented with 3 weeks of pain, numbness, and progressive weakness of the right lower back and lower extremity associated with the appearance of red lesions. He was on tacrolimus, azathioprine, and prednisone for immunosuppression due to a history of heart transplantation 3 years prior. He denied headache, neck stiffness, fever, confusion, or bowel or bladder incontinence. Physical examination was notable for the absence of a cardiac murmur, 2/5 right lower extremity weakness, and painless purpuric macules scattered along the anterior and posterior regions of his right buttock and right lower extremity in a nondermatomal distribution without vesicles (Figure 1).

**Figure 2:**
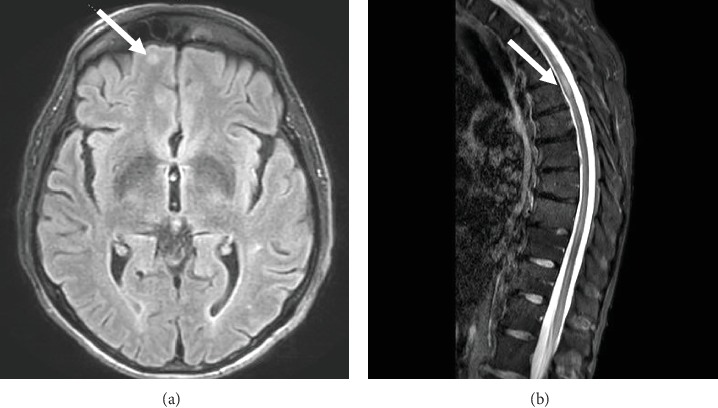
MRI with intravenous contrast of the brain and spine showing subacute infarcts in the bilateral cerebral hemispheres and left cerebellar hemisphere (a) and an enhancing lesion within the posterior aspect of the right hemicord at the T5 level (b).

**Figure 3:**
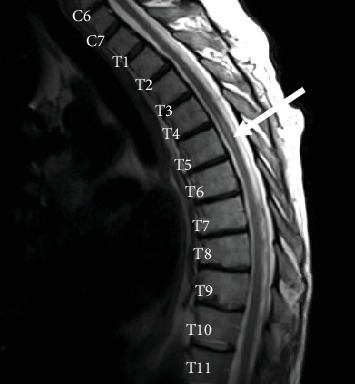
Repeat MRI with intravenous contrast of the spine demonstrating an increased T2 signal at the T5-T6 level of the spinal cord consistent with infarct.

Laboratory testing was negative for human immunodeficiency virus, hepatitis, syphilis, antineutrophil cytoplasmic antibody, and cryoglobulinemia. Complement levels were normal. Blood cultures revealed no growth, and echocardiography did not reveal valvular vegetations. Pathology from a biopsy of the rash revealed an inflammatory infiltrate of lymphocytes, neutrophils, some eosinophils, and nuclear dust with extravasation of erythrocytes. This was consistent with leukocytoclastic vasculitis. Magnetic resonance imaging (MRI) with intravenous contrast of the brain and spine was notable for subacute infarcts in the bilateral cerebral hemispheres and left cerebellar hemisphere (Figure 2(a)) and an enhancing lesion within the posterior aspect of the right hemicord at the T5 level (Figure 2(b)). Due to suspicion for an atypical VZV infection given the constellation of findings, intravenous acyclovir was initiated. Lumbar puncture was performed, and VZV was detected by qualitative real-time polymerase chain reaction (PCR) in the cerebrospinal fluid. The assay used PCR primers and probes that specifically target the glycoprotein B gene of VZV with the lower limit of detection being 250 copies/mL. The patient was treated with intravenous acyclovir for 14 days (10 mg/kg three times daily), followed by valacyclovir (400 mg by mouth twice daily) with a plan for 3 months of oral therapy. His maintenance immunosuppressive therapy for heart transplantation was reduced. His rash and pain resolved, and he was discharged home with some improvement in his motor function. However, 9 days after discharge, he presented again to the emergency room with an acute onset of left leg weakness in the absence of rash and was found to have a new spinal cord infarct at the T6 level on MRI (Figure 3). He was treated once again with intravenous acyclovir but the course was extended to 21 days and was followed by valacyclovir 400 mg twice daily for lifelong therapy. He improved clinically and has not had further recurrence since then.

## 3. Discussion

VZV vasculopathy is associated with significant morbidity and mortality when treatment is delayed. Therefore, rapid diagnosis is essential. Interestingly, our patient did not present with the classic vesicular rash of VZV but rather with cutaneous vasculitis, which is often painless, thought to be a rare manifestation of VZV infection, and reported as preceding the classic vesicular rash [3–5]. In our patient's case, a high degree of clinical suspicion was needed to make the diagnosis. Other considerations in the differential diagnosis for this patient with multifocal infarcts included endocarditis with septic emboli, HIV-associated vasculitis, syphilis, cryoglobulinemia syndrome, and autoimmune inflammatory forms of vasculitis. However, in our patient's case, the diagnosis of VZV vasculopathy was supported by laboratory, radiographic, and cerebrospinal fluid abnormalities [6, 7].

Although most patients with VZV vasculopathy have an abnormal MRI, the findings are not necessarily diagnostic. Additionally, a serum VZV IgG test is of little value, because it is suggestive of immunization or prior exposure to the virus and does not localize the VZV infection to the central nervous system (CNS). Therefore, confirmation of the diagnosis requires sampling of cerebrospinal fluid and detection of VZV DNA by real-time polymerase chain reaction or anti-VZV IgG antibody by enzyme immunosorbent assay [7, 8]. Both tests are highly specific for the diagnosis, although the anti-VZV IgG antibody is more sensitive. Importantly, the absence of cerebrospinal fluid pleocytosis does not exclude the disease, and a negative VZV PCR alone is not sufficient to exclude the diagnosis, as this requires both the PCR and antibody test to be negative [7, 8].

The presence of VZV DNA in the cerebrospinal fluid is most commonly assessed by qualitative PCR methods with a detection limit ranging from 50 to 250 copies/mL, which is similar to what was used in our case, although quantitative real-time PCR detection of VZV DNA has also been described [2, 9, 10]. Treatment of VZV vasculopathy is based primarily on expert opinion [2, 8]. Since the underlying process is viral infection of the arteries, intravenous acyclovir for 14 days is the treatment of choice. Oral acyclovir has relatively poor bioavailability. Some experts advocate the use of an oral steroid (e.g., prednisone) at a dose of 1 mg/kg for the first 5 days of treatment or if there is lack of clinical improvement on IV antiviral therapy. For recurrent disease, prolonged therapy with IV acyclovir for 21 days followed by oral valacyclovir for 1-2 months is recommended.

In conclusion, we present a rare case of VZV vasculopathy that manifested with both cerebral and spinal infarctions and cutaneous vasculitis without the appearance of the classic vesicular rash. We believe that this is an important case to share with the medical community given that such an atypical manifestation may result in substantial morbidity without prompt identification and treatment.
